# Biological activities of *Centaurium erythraea* and its main chemotypes: menthol, carvacrol, and tricosane

**DOI:** 10.1186/s13568-025-01956-9

**Published:** 2025-10-21

**Authors:** Tarik Aanniz, Nasreddine El Omari, Saad Bakrim, Abdelaali Balahbib, Hamza Elhrech, Taoufiq Benali, Mohammed Amanullah, Long Chiau Ming, Said Moshawih, Abdelhakim Bouyahya

**Affiliations:** 1 Medical Biotechnology Laboratory, Rabat Medical & Pharmacy School, Mohammed V University in Rabat, B.P. 6203 Rabat, Morocco; 2 High Institute of Nursing Professions and Health Techniques of Tetouan, Tetouan, Morocco; 3 Geo-Bio-Environment Engineering and Innovation Laboratory, Molecular Engineering, Biotechnology and Innovation Team, Polydisciplinary Faculty of Taroudant, Ibn Zohr University, 80000 Agadir, Morocco; 4 High Institute of Nursing Professions and Health Techniques of Errachidia, Errachidia, Morocco; 5 Laboratory of Human Pathologies Biology, Department of Biology, Faculty of Sciences, Mohammed V University in Rabat, 10106 Rabat, Morocco; 6 Laboratory of Ecotoxicology, Bioresources, and Coastal Geomorphology, Polydisciplinary Faculty of Safi, Cadi Ayyad University, PO Box 4162, 46000 Safi, Morocco; 7 Department of Clinical Biochemistry, College of Medicine, King Khalid University, Abha, Kingdom of Saudi Arabia; 8 Datta Meghe College of Pharmacy, Datta Meghe Institute of Higher Education and Research (Deemed to Be University), Sawangi (M), Wardha, India; 9 Faculty of Medical and Life Sciences, Sunway University, Sunway City, Malaysia; 10 Department of Pharmaceutical Sciences, Faculty of Pharmacy, Al-Ahliyya Amman University, Amman, Jordan

**Keywords:** *Centaurium erythraea*, Essential oil, In vitro, In silico, Biological activity, Carvacrol, Menthol, Tricosane

## Abstract

Here, we investigated the antioxidant, antidiabetic, dermatoprotective, and antibacterial capacities of *Centaurium erythraea* essential oil (CEEO) and its major compounds namely menthol, carvacrol, and tricosane. The main findings revealed that CEEO, menthol, and tricosane impeded the proliferation of Gram-positive bacteria more efficiently than that of Gram-negative bacteria. The most active molecules in the FRAP assay were carvacrol (59.96 ± 2.60 µg/mL) and menthol (57.93 ± 15.63 µg/mL), while the DPPH and ABTS tests demonstrated a potent activity of CEEO. For the antidiabetic tests, CEEO displayed significantly lower IC_50_ values, 168.62 μg/mL for α-amylase and 87.18 μg/mL for α-glucosidase. These values indicated a marked inhibition compared to menthol, carvacrol, and tricosane as they exhibited a moderate inhibition of α-amylase (IC_50_ = 471.50 μg/mL, IC_50_ = 566.20 μg/mL, and IC_50_ = 385.26 μg/mL, respectively) and α-glucosidase (183.25 μg/mL, 201.18 μg/mL, 192.30 μg/mL, respectively). With an IC_50_ of 41.863 ± 0.031 μg/mL, CEEO also showed excellent efficacy against tyrosinase. For the inhibition of elastase, similar IC_50_ values for CEEO (113.02 ± 3.37 μg/mL) and menthol (112.06 ± 3.70 μg/mL) were reported while those of carvacrol and tricosane were 124.68 ± 4.31 and 145.26 ± 4.62 μg/mL, respectively. To sum up, CEEO, carvacrol, menthol, and tricosane exert various biological activities that need to be deeply investigated and optimized to select the best compound and/or combination.

## Highlights

Main compounds of Centaurium erythraea L., essential oils, menthol, carvacrol, and tricosane, showed important antimicrobial and anti-enzymatic effects.

Menthol is responsible for the dermatoprotective activity of CEEO.

Tricosane and carvacrol revealed the most prominent antibacterial and antidiabetic effects.

**Graphical abstract:**

## Graphical abstract

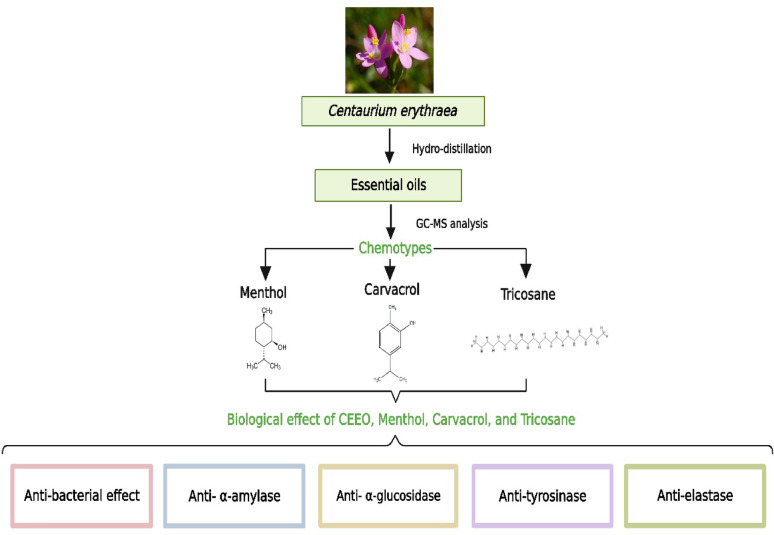

## Introduction

Natural products have been the foundation of traditional healing worldwide and an important component of history, civilization, and culture (Khan et al. 2022; Nasim et al. 2022; Nguyen-Vo et al. 2020; Veeresham 2012). Plant products were used historically as herbal medicinal preparations with hundreds of substances until the nineteenth century, when pure compounds started to be isolated and characterized. This motivated researchers to separate and purify the key molecule(s) guaranteeing the observed efficiency of the plant extracts. Many substances such as ajoene, camphene, carvone, chalcones, chrysoeriol, grifoline, hispolon, pinosylvine, and quercetin, have been extensively investigated (Aanniz et al. 2024; Bakrim et al. 2022, 2024; Bouyahya et al. 2023, 2025; El Hachlafi et al. 2024; Hachlafi et al. 2023; Nasim et al. 2022; Touhtouh et al. 2023, 2024). Besides, computational methods allow the processing of complicated natural products and the use of their structures to create novel derivatives (Purohit et al. 2024). Moreover, the identification of the most active compounds provides the opportunity to combine several single-component treatments to avert the development of resistance or to target several pathological processes at once (Wang et al. 2023).

Essential oils (EOs) are intricate substances consisting of numerous components with significant variations in composition. Hence, it becomes challenging to ascertain the specific pathways targeted within the body. The composition of EOs might significantly differ across various producers and even within the same manufacturer, which could negatively affect the efficiency of EOs. The variation, both quantitatively and qualitatively, of the EO could be ascribed to various elements such as the as cultivar/species (Ramsey et al. 2020), harvest season (Russo et al. 2013), latitude (El-Jalel et al. 2018), geographical origin (Song et al. 2023; Tammar et al. 2019), stage of development (Bouyahya et al. 2020a, b), and extraction process (Bozova et al. 2024; Gölükcü et al. 2024). Nowadays, EOs and their main constituents are garnering more attention due to their relatively mild characteristics, their comparatively safe characteristics, widespread consumer acceptance, and potential for diverse uses, hence requiring further investigation (Martínez et al. 2018; Tammar et al. 2019). Researchers are investigating the integration of EOs and their components as natural antioxidants and antibacterial agents to prolong food shelf life (Ben Jemaa et al. 2018a, b; Jemaa et al. 2018; López-Meneses et al. 2015; Russo et al. 2013). EOs are garnering considerable attention in the food business owing to their statuts as Generally Recognized As Safe (GRAS) (Ben Jemaa et al. 2018a, b), and many EOs have received approval for use, especially in the United States (Ben Jemaa et al. 2018a, b; Pandey et al. 2016; Regnault-Roger et al. 2012).

A rigorous and systematic investigation of Moroccan aromatic and medicinal plants has become imperative. *Centaurium erythraea* (*C. erythraea*) is an annual or biennial herbaceous plant widely used to prevent and to manage various diseases, i.e., fever, dyspeptic disorders, diabetes, loss of appetite, digestive disorders, atopic dermatitis, cancer, pneumonia, cardiovascular diseases, asthma, gastric pain, kidney and skin diseases (El Menyiy et al. 2021). *C. erythraea* essential oil (CEEO) and extract exert many valuable activities, i.e., antibacterial, antioxidant, antifungal, antileishmanial, cytotoxic, antidiabetic, anti-inflammatory, insecticidal/larval development inhibition, spasmolytic, gastroprotective, dermatoprotective, hepatoprotective, neuroprotective, analgesic, and antipyretic properties (Bouyahya et al. 2017; Bouyahya et al. 2019a, b; Božunović et al. 2018; El Menyiy et al. 2021; Guedes et al. 2019; Šiler et al. 2014). In the previous study of our team, Bouyahya et al. found that CEEO contains around forty components and highlighted certain compositional differences resulting in different biological capacities from three developmental stages examined (Bouyahya et al. 2019a, b). However, the effect of the main compounds has not yet been evaluated to correlate the effect of CEEO with its main chemotypes. Hence, our objective is to analyze the chemical composition of CEEO at the flowering stage, focusing on its main constituents (carvacrol, menthol, and tricosane) to assess their antioxidant, antidiabetic, antibacterial, and dermatoprotective properties.

## Materials and methods

### Chemicals and reagents

We purchased the compounds menthol, carvacrol, tricosane, quercetin, and acarbose from Sigma-Aldrich (France). The α-glucosidase from *Saccharomyces cerevisiae* and the α-amylase from *Bacillus licheniformis* were also obtained from reliable commercial sources. Additional reagents were carefully selected from reputable suppliers. Mueller–Hinton agar was sourced from Biokar in Beauvais, France. All other chemicals used are of analytical grade.

### Collection of plant material

*C. erythraea* was collected from its natural habitat in the Ouezzane province of northwest Morocco (34° 47′ 50′′ N and 5° 34′ 56′′ W) in October 2016, confirmed verified at the Scientific Institute of Rabat (SIR) (Morocco), and a voucher sample (RAB30) has been archived for reference. The harvested plant was air-dried in a shaded area at room temperature.

### Essential oil isolation

The CEEO was extracted using a Clevenger-type apparatus. Subsequently, the obtained CEEO was desiccated with anhydrous sodium sulfate and then stored at 4 °C for further studies.

### Chemical composition determination

Our previous publication (Bouyahya et al. 2019a, b) determined and quantified the volatile compounds of CEEO.

### Antibacterial activity

The antibacterial activity of CEEO, together with that of carvacrol, menthol, and tricosane, was evaluated by the disk diffusion method (Benali et al. 2020a, b; Bouyahya et al. 2019a, b). The strains *Escherichia coli* (ATCC 25922), *Pseudomonas aeruginosa* NIH, *Listeria monocytogenes* (ATCC 13932), and *Staphylococcus aureus* (ATCC 29213) were used. After overnight incubation at 30 °C, the inhibition zone diameters (IZD) were measured in millimeters (mm). Next, the microdilution method served to assess the minimum inhibitory concentration (MIC) and minimum bactericidal concentration (MBC) (Bouyahya et al. 2020a, b).

### Antioxidant activity assays

#### DPPH test

This assay used DPPH radical to assess the free radical-scavenging ability. Samples dissolved in methanol were mixed with a methanolic solution of DPPH and incubated, in triplicate, at ambient temperature for 30 min (El Omari et al. 2023). Absorbance was read at 517 nm, and the percentage of DPPH radical scavenging and the inhibition in IC_50_ (μg/mL) were calculated. Trolox served as a positive control.

#### Reducing ferric power test

CEEO, carvacrol, menthol, and tricosane were mixed with phosphate buffer and potassium ferricyanide solution, incubated, and treated with trichloroacetic acid. After centrifugation, the upper layer was analyzed for absorbance (700 nm) with Trolox serving as a reference. The assay was done in triplicate, and the IC_50_ (μg/mL) was calculated (El Omari et al. 2023).

#### ABTS radical scavenging activity

The ABTS cation radical was generated from the reaction between ABTS solution and potassium persulfate with Trolox as a positive control. The assay was performed in triplicate and expressed in IC_50_ (μg/mL) (El Omari et al. 2023).

### Antidiabetic activity

#### α-Amylase inhibitory test

We investigated the possible inhibition of the α-amylase enzyme by various concentrations of CEEO, carvacrol, menthol, and tricosane. Initially, 250 μL of each sample was mixed with 250 μL of sodium phosphate buffer (SPB) (0.02 M & pH = 6.9) and α-amylase enzyme (240 U/mL). The blend was then incubated at 37 °C/20 min. Then, a 1% starch solution (250 μL) in SPB was added, followed by incubation at 37 °C/15 min. Thereafter, 1 mL of dinitrosalicylic acid (DNS) was also added, and the mix was heated for 10 min (Bouyahya et al. 2019a, b; Omari et al. 2023). The solution was then diluted with 2 mL of sterile water, and the OD (540 nm) was measured. For result accuracy, acarbose, a known effective α-amylase inhibitor, served as a reference. The IC_50_ value represents the concentration of the α-amylase inhibitor required to inhibit 50% of activity. The % of inhibition was calculated using the formula:

$$ \begin{gathered} \% {\text{ of inhibition}} \hfill \\ {\text{ }} = \left( {1 - \frac{{\left( {{\text{Abs enz}} + {\text{sub }} - {\text{ Abs sub}}} \right) - \left( {{\text{Abs sample }} - {\text{ Abs control}}} \right)}}{{\left( {{\text{Abs enz}} + {\text{sub }} - {\text{ Abs sub}}} \right)}}} \right){\mkern 1mu} \times {\mkern 1mu} 100. \hfill \\ \end{gathered} $$

#### α-Glucosidase inhibitory test

The inhibition of α-glucosidase action was assessed using the pNPG substrate as previously detailed (Bouyahya et al. 2021). A mixture of 200 μL of the samples and 100 μL SPB (0.1 M & pH = 6.7), mixed with α-glucosidase (0.1 U/mL), underwent incubation at 37 °C/10 min. Next, 200 μL of pNPG (1 mM) solution in SPB (0.1 M & pH = 6.7) was added, then incubated at 37 °C/30 min. The measurement of α-glucosidase inhibition was done by measuring the OD (405 nm) after adding 1 mL of Na_2_CO_3_ (0.1 M). The efficacy was denoted as % of inhibition, and the IC_50_ values were determined with acarbose as a reference.

### Dermatoprotective capacity

#### Tyrosinase inhibitory assay

Following the method described by Marmouzi et al., the inhibitory ability of CEEO, carvacrol, menthol, and tricosane was used to assess their dermatoprotective impact (Marmouzi et al. 2019). In summary, 25 µL of sample was mixed with 100 µL of a tyrosinase solution (333 U/mL in 50 mM SPB, pH 6.5) and incubated for 10 min at 37 °C. After that, 300 µL of L-DOPA (5 mM) was added, and the mixture was kept at 37 °C for 30 min, and the absorbance was recorded at 510 nm. Tyrosinase inhibition rates were computed for EO dosages of 40, 60, 120, and 160 μg/mL, and IC_50_ values were determined with quercetin serving as a reference.

#### Elastase inhibitory assay

To perform the experiment, varying amounts of CEEO, carvacrol, menthol, and tricosane were dissolved in methanol at concentrations of 0.5, 1, 2, and 3 mg/mL. Then, 200 µL of an elastase solution prepared in Tris–HCl buffer (0.2 M, pH 8.0) was mixed with 50 µL of each sample. Following a 15-min incubation at 25 °C, 200 µL of *N*-succinyl-Ala-Ala-p-nitroanilide solution was added, and the reaction mixtures were homogenized. Absorbances were recorded at 410 nm after an additional 20-min incubation at 25 °C. The data obtained were used to determine the IC_50_ and to calculate the percent inhibition of elastase. Quercetin was used as a positive control to validate experimental design (Jeddi et al. 2023).

#### Molecular docking analysis

Molecular docking is an efficient in silico option used to understand the binding mode between molecular structures and protein targets responsible for biological activity. Molecular docking simulations were carried out using the AutoDock Vina v 1.1.2 (Mazouri et al. 2021; Touhtouh et al. 2023). All ligands were exported from the PubChem database. The 3D structures of acetylcholinesterase (AChE; ID: 1c2b), butyrylcholinesterase (BChE; ID: 4bds), α-amylase (ID: 4w93), α-glucosidase (ID: 2ze0), tyrosinase (ID: 5i38), and elastase (ID: 1bru) were obtained from the Protein Data Bank (https://www.rscb.org), Grid dimensions were set to (48 Å × 50 Å × 56 Å), (52 Å × 70 Å × 52 Å), (40 Å × 26 Å × 42 Å), (44 Å × 48 Å × 46 Å), (44 Å × 58 Å × 46 Å), and (20 Å × 18 Å × 28 Å), respectively. The optimal poses of ligands were analyzed for interactions using Discovery Studio 2024 (BIOVIA).

#### ADMET prediction

The physiochemical properties and drug-likeness of the major compounds were assessed to predict their pharmacokinetic properties (Rodrigues et al. 2020). The SMILES structures of the main compounds were taken from the PubChem database and entered into the pkCSM ADMET descriptor algorithm (https://biosig.lab.uq.edu.au/pkcsm/) (Pires et al. 2015).

#### Statistical analysis

All antimicrobial, antioxidant, antidiabetic, and dermatoprotective assays were performed in triplicate to ensure the repeatability and reliability of the results. Data are presented as mean ± standard deviation (SD). Statistical analysis was conducted using GraphPad Prism (version 10.1). A two-way ANOVA followed by Tukey’s multiple comparison test was used to determine statistically significant differences between groups.

## Results

### Chemical composition of CEEO

Our previous study (Bouyahya et al. 2019a, b) determined 37 compounds at the flowering stage. They belong to eight classes, i.e., sesquiterpene hydrocarbons, monoterpene hydrocarbons, oxygenated sesquiterpenes, oxygenated monoterpenes, ketones, acids, aldehydes, and alkanes (Table 1). During this stage, oxygenated monoterpenes accounted for 44.10% of the chemicals, followed by alkanes (16.49%) and sesquiterpene hydrocarbons (8.15%). It appears that the three main chemotypes are menthol (20.82%), tricosane (15.27%), and carvacrol (8.73%). However, other chemicals were also found, including isomenthone (3.09%), decanal (3.31%), piperitone (2.58%), hexadecane (2.21%), germacrene D (2.17%), camphor (2.13%), linalool (1.94%), and menthone (1.82%).

**Table 1:** Major bioactive compounds of *C. erythraea* essential oils identified by (Bouyahya et al. 2019a, b)

R_I_	Compounds	CEEO	ID
1171	Menthol	20.82%	MS, Ri
1340	Carvacrol	8.73%	MS, Ri
1771	Tricosane	15.27%	MS, Ri

CEEO: *C. erythraea* essential oil; ID: identification; MS: mass spectra; Ri: retention index

### Antibacterial activity

First, agar diffusion assays were performed using two Gram-negative strains (*E. coli* and *P. aeruginosa*) and two Gram-positive strains (*S. aureus* and *L. monocytogenes*) (Table 2). Regarding the effect of CEEO, the Gram-positive strain *S. aureus* was more sensitive, followed by *L. monocytogenes*. As for Gram-negative, *P. aeruginosa* showed no sensitivity, while no effect was observed towards *E. coli*. Of the three compounds tested, carvacrol was the most potent one, with an IZD of more than 30 mm against all four strains tested. Both menthol and tricosane were effective against Gram-positive bacteria (IZD > 29 mm) but showed less effectiveness against Gram-negative bacteria (IZD < 18.5 mm). High values of MIC (0.25–2 µg/mL) and MBC (1–2 µg/mL) were shown for CEEO, which corroborates the findings from agar diffusion tests. The MIC and MBC values supported the strength of carvacrol shown in the agar diffusion results since the lowest MIC being 0.0125 µg/mL and the MBC at 0.025 µg/mL against *S. aureus*. The MBC/MIC of carvacrol against *P. aeruginosa* and *L. monocytogenes* was equal to 1, suggesting a bactericidal effectiveness. For menthol, the MIC was between 0.25 and 0.5 µg/mL, and the MBC was between 0.5 and 2 µg/mL. For tricosane, MIC ranged from 0.125 to 0.5 µg/mL and MBC from 0.5 to 1 µg/mL with potential bactericidal effect toward *P. aeruginosa* and *L. monocytogenes* (MBC/MIC = 1).

**Table 2:** IZD (mm), MIC (µg/mL), and MBC (µg/mL) of the major chemotypes of CEEO

Molecules		Gram-negative		Gram-positive	
		*E. coli*	*P. aeruginosa*	*S. aureus*	*L. monocytogenes*
CEEO	MIC	1	2	0.25	0.25
	MBC	1	> 2	1	1
	IZD	NA	11 ± 0.5^d^	28 ± 1.5^a^	26 ± 2.5^b^
Menthol	MIC	0.5	0.25	0.5	0.25
	MBC	2	1	1	0.5
	IZD	14 ± 2^a^	18 ± 4.5^b^	32 ± 2.64^a^	26.33 ± 1.52^b^
Carvacrol	MIC	0.125	0.5	0.0125	0.125
	MBC	0.25	0.5	0.025	0.125
	IZD	31.33 ± 2.51^b^	29 ± 2.64^c^	35.33 ± 1.52^a^	33 ± 2.64^a^
Tricosane	MIC	0.25	0.5	0.125	0.25
	MBC	1	0.5	0.5	0.25
	IZD	20.67 ± 2.08^C^	17 ± 2.51^b^	29 ± 4.58^a^	31.67 ± 4.04^b^
Chloramphenicol	MIC	8	64	8	16
	MBC	32	64	32	32

NA: no activity; IZD: inhibition zone diameter; MIC: minimum inhibitory concentration; MBC: minimum bactericidal concentration; Different superscript letters in the same column indicate statistically significant differences (*p* < 0.05), whereas identical letters indicate no significant difference

### Antioxidant activity

**Fig. 1:**
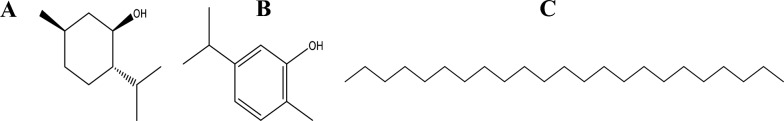
Chemical structures of three main compounds of CEEOs. **A** Menthol, **B** Carvacrol, **C** Tricosane

The in vitro antioxidant potential was investigated on the basis of radical scavenging of DPPH and ABTS, while reducing activity was assessed by FRAP, with Trolox as a control (Fig. 1**; **Table 3). CEEOs and the pure molecules demonstrated marked potential to scavenge DPPH free radicals, with IC_50_ values of 47.18 ± 3.62 μg/mL (CEEO), 48.06 ± 4.31 μg/mL (carvacrol), 58.89 ± 4.03 μg/mL (menthol), and 91.03 ± 0.3 μg/mL (tricosane). Remarkably, CEEO was found to have a stronger antioxidant effect than tricosane, carvacrol, and menthol. In addition, the results of the antioxidant activity of CEEO and carvacrol were close to those of Trolox (IC_50_ of 34.12 ± 2.13 μg/mL). The ABTS assay revealed that CEEO (53.25 ± 2.19 µg/mL) exhibited a similar value as compared to Trolox (55.25 ± 4.19 µg/mL), while menthol (71.76 ± 3.16 µg/mL) and carvacrol (81.41 ± 4.31 µg/mL) exerted moderate activities. In the FRAP assay, menthol (57.93 ± 15.63 µg/mL) and carvacrol (59.96 ± 2.60 µg/mL), as well as CEEO (65.34 ± 3.71 µg/mL), were the most powerful, with values similar to those of Trolox. On the other hand, tricosane was the least effective one in all three tests (DPPH: 91.03 ± 0.3 µg/mL; FRAP: 103.9 ± 2.48 µg/mL; ABTS: 120.34 ± 2.97 µg/mL).

**Table 3:** Antioxidant properties of CEEO, menthol, carvacrol, tricosane, and Trolox (IC_50_ in µg/mL)

	DPPH	ABTS	FRAP
CEEO	47.18 ± 3.62^h^	53.25 ± 2.19^b^	65.34 ± 3.71^f^
Menthol	58.89 ± 4.03^a^	71.76 ± 3.16^a^	57.93 ± 15.63^f^
Carvacrol	48.06 ± 4.31^h^	81.41 ± 4.31^b^	59.96 ± 2.60^f^
Tricosane	91.03 ± 0.3^b^	103.9 ± 2.48^c^	120.34 ± 2.97^a^
Trolox	34.12 ± 2.13^h^	55.25 ± 4.19^b^	54.74 ± 3.85^f^

Values sharing the same letter within a column or row are not significantly different (*p* > 0.05), while those with different letters are significantly different (*p* < 0.05)

### Enzyme inhibitory activity

The anti-enzymatic effects of CEEO and its major compounds were evaluated in the context of metabolic (α-amylase, and α-glucosidase) and skin disorders (tyrosinase, and elastase).

### Antidiabetic activity

**Fig. 2:**
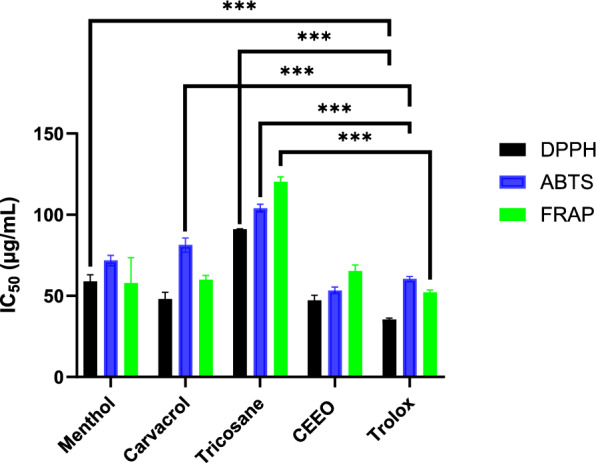
Antioxidant activity (IC_50_ in µg/mL) of CEEO and the three compounds compared to Trolox

The data presented measures the inhibitory capacity of the CEEO and its main chemotypes toward α-amylase and α-glucosidase in terms of IC_50_ (μg/mL) (Fig. 2**, **Table 4). The application of CEEO on α-amylase resulted in an IC_50_ value of 168.62 ± 0.636 μg/mL, compared to acarbose’s IC_50_ value of 396.42 ± 4.83 μg/mL, showing that CEEO is much more effective at inhibiting this enzyme. Moreover, tricosane showed an IC_50_ value (385.26 ± 4.64 μg/mL) similar to that of acarbose, indicating a promising effect. However, IC_50_ values of menthol (471.50 ± 3.93 μg/mL) and carvacrol (566.20 ± 5.19 μg/mL) were higher than acarbose, meaning a moderate to weak effect. The strongest ability to hinder the α-glucosidase, activity was shown by CEEO at IC_50_ value 87.18 ± 0.422 μg/mL, which is better than acarbose (199.53 ± 3.26 μg/mL). Additionally, menthol, carvacrol, and tricosane (183.25 ± 2.35, 201.18 ± 5.40, and 192.30 ± 3.55, respectively) showed similar acarbose IC_50_ value, suggesting good inhibition capacities.

**Table 4:** Enzyme inhibitory activity of the major chemotypes of *C. erythraea* (IC_50_ as μg/mL)

	α-Amylase	α-Glucosidase	Tyrosinase	Elastase
CEEO	168.62 ± 0.636^a^	87.18 ± 0.422^b^	41.863 ± 0.031^d^	113.02 ± 3.37^c^
Menthol	471.50 ± 3.93^b^	183.25 ± 2.35^f^	270.21 ± 4.96^a^	112.06 ± 3.70^d^
Carvacrol	566.20 ± 5.19^c^	201.18 ± 5.40^d^	302.48 ± 6.43^b^	145.26 ± 4.62^a^
Tricosane	385.26 ± 4.64^d^	192.30 ± 3.55^f^	45.70 ± 2.24^c^	124.68 ± 4.31^b^
Acarbose	396.42 ± 4.83^d^	199.53 ± 3.26^f^	–	–
Quercetin	–	–	246.90 ± 2.54^f^	9.08 ± 0.21^h^

Values sharing the same letter within a column or row are not significantly different (*p* > 0.05), while those with different letters are significantly different (*p* < 0.05)

### Dermatoprotective effects

**Fig. 3:**
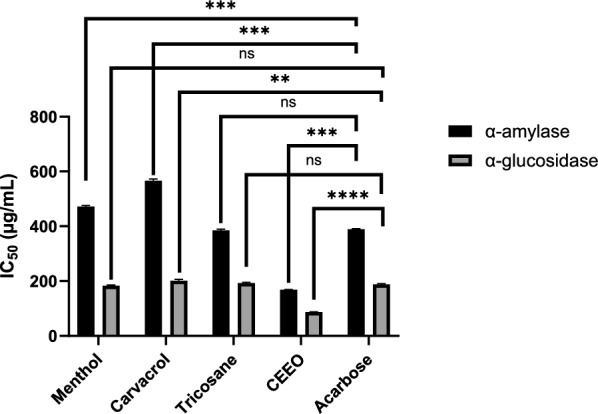
antidiabetic activity (IC50 in µg/mL) of CEEO and the three compounds compared to Acarbose. ns: not significant

The main findings related to the tyrosinase inhibition expressed in terms of IC_50_ showed important variations. Compared to quercetin (IC_50_ = 246.90 ± 2.54 μg/mL), CEEO and tricosane showed much stronger anti-tyrosinase effect, with IC50 values of 41.863 ± 0.031 and 45.70 ± 2.24 μg/mL, respectively. In contrast, menthol (IC_50_ of 270.21 ± 4.96 μg/mL) and carvacrol (IC_50_ of 302.48 ± 6.43 μg/mL) exhibited relatively lower activity, although it is still stronger than that of quercetin. In contrast, the results of the elastase inhibitory results indicated that all the tested compounds and CEEO had low activity (IC50 values between 112.06 ± 3.7 and 145.26 ± 4.62 μg/mL) when compared to quercetin (9.08 ± 0.21 μg/mL) (Fig. 3**, **Table 4).

### Molecular docking analysis

**Fig. 4:**
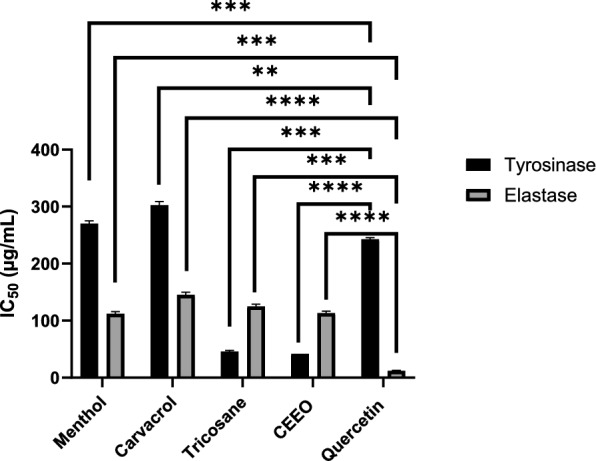
Dermatoprotective effects (IC_50_ in µg/mL) of CEEO and the three compounds compared to Quercetin

**Fig. 5:**
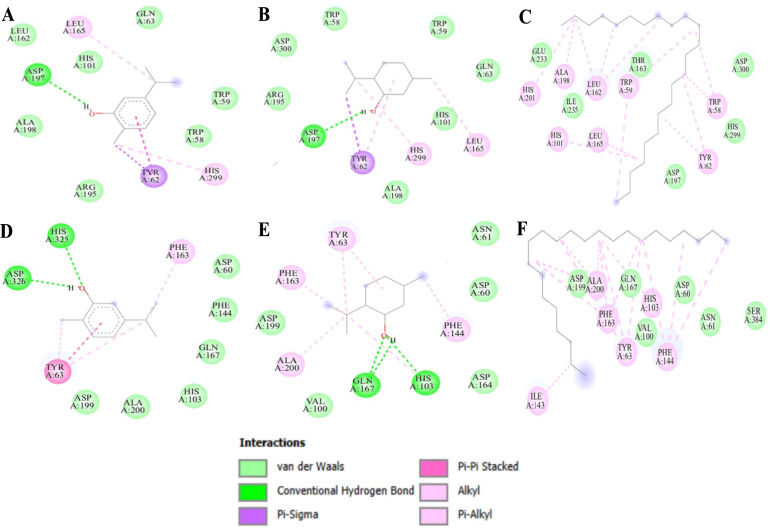
Protein–ligand interaction: **A** α-amylase-Carvacrol; **B** α-amylase-Menthol; **C** α-amylase-Tricosane; **D** α-glucosidase-Carvacrol; **E** α-glucosidase-Menthol; **F** α-glucosidase-Tricosane

**Fig. 6:**
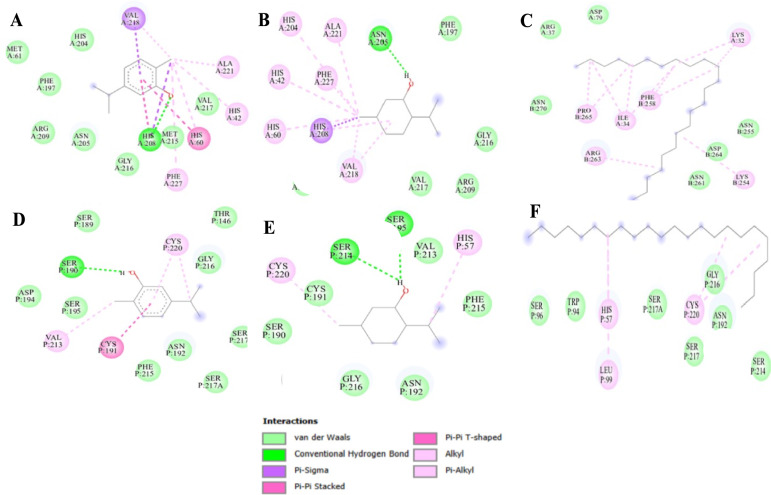
Protein–ligand interaction: **A** Tyrosinase-Carvacrol; **B** Tyrosinase-Menthol; **C** Tyrosinase-Tricosane; **D** Elastase-Carvacrol; **E** Elastase-Menthol; **F** Elastase-Tricosane

To understand the obtained results in the enzyme inhibition tests, the main EO compounds were docked into the active sites of the four targets (α-amylase, α-glucosidase, elastase, and tyrosinase) and achieved acceptable binding scores upon docking (Fig. 4, Table 5**)**. For the docking results with α-amylase, carvacrol had the best binding score of − 6.3, followed by menthol (− 5.7 kcal/mol) and tricosane (− 5.0 kcal/mol). Carvacrol interacted with the residue ASP197 through a conventional hydrogen bond, engaged TYR62 via pi-sigma interactions, and formed pi-alkyl bonds with HIS299 and LEU165. Additionally, Van der Waals forces were observed with ALA198, LEU162, HIS101, ARG195, TRP58, and TRP59. For α-glucosidase, the binding scores of carvacrol, menthol, and tricosane were − 6.4, − 6.4 and − 5.0 kcal/mol, respectively. Carvacrol interacted with the residue ASP326 via a conventional hydrogen bond. In the catalytic site, carvacrol made more connections with HIS325 (hydrogen bond), TYR63 (pi–pi stacked bond), and PHE163 (pi-alkyl bond). On the other hand, menthol interacted with residues GLN167, HIS103, PHE144, ALA200, PHE163, and TYR63 via pi-alkyl bonds. Also, when looking at how this compound interacts with both α-amylase and α-glucosidase compared the redocked scores of acarbose (used as a standard), carvacrol showed the best binding score for both enzymes, while tricosane had lower scores. In the context of elastase docking, carvacrol, menthol, and tricosane had weaker binding affinities with scores of − 4.7, − 4.5, and − 3.6 kcal/mol, respectively, while quercetin had a stronger score of − 7.1 kcal/mol. When analyzing Fig. 5, it was found that carvacrol interacts with elastase residues through conventional hydrogen bonds with SER190, pi-alkyl bonds with CYS220 and VAL213, and Van der Waals forces with ASP194, SER195, SER189, THR146, GLY216, ASN192, and PHE215. Regarding tyrosinase docking, carvacrol had the best binding energy (− 6.6 kcal/mol), surpassing that of menthol and tricosane (− 5.2 and − 4.0 kcal/mol, respectively), when compared to quercetin. Carvacrol interacted with tyrosinase through hydrogen bonds with HIS208, pi-sigma bonding with VAL218, and pi-alkyl interactions involving HIS42, PHE227, and ALA221. Additionally, Van der Waals forces were observed with HIS204, PHE197, ARG209, ASN205, VAL217, and MET217. Overall, carvacrol emerges as the most potent compound, demonstrating strong binding energy affinity to the targeted enzymes, primarily through conventional hydrogen bonds within their active sites (Fig. 6).

**Table 5:** Docking study results for six targets enzymes binding energy affinities (Kcal/mol)

	α-Amylase	α-Glucosidase	Elastase	Tyrosinase
Carvacrol	− 6.3	− 6.4	− 4.7	− 6.6
Menthol	− 5.7	− 6.4	− 4.5	− 5.2
Tricosane	− 5.0	− 5.0	− 3.6	− 4.0
Galantamine*				
Acarbose*	− 7.0	− 9.3		
Quercetin*			− 7.1	− 8.1

*Standards

### ADMET properties of major chemotypes

The *in-silico* prediction of the ADME-Tox properties of a compound leads to gathering more detailed safety information. The ADMET properties of carvacrol, menthol, and tricosane show different behaviors in how they are processed in the body and their safety, reflecting their specific pharmacokinetic and pharmacodynamic traits (Table 6). All three chemicals have a high intestinal absorption rate in humans (> 90%), with menthol having the highest rate (96.5%). However, compared to menthol and carvacrol, tricosane exhibits a much lower water solubility (log − 7.539), which could affect its bioavailability. All three molecules have similar and advantageous Caco-2 permeability. Interestingly, all the compounds are P-glycoprotein substrates, indicating that they may have an impact on efflux mechanisms. However, none of them inhibit P-gp I, except for tricosane, which inhibits P-gp II. Tricosane has the highest volume of distribution (log VDss 1.206), which suggests that it penetrates tissues more deeply. It is also fully protein-bound (Fu = 0), which may limit the amount of the free circulating fraction. While its CNS permeability (log PS = − 0.37) is still moderate, all compounds can cross the BBB well (log BBa > 0), with tricosane showing the best ability (1.173). All the compounds. except for tricosane, do not get broken down by the two main liver enzymes, CYP2D6 and CYP3A4, while tricosane is affected by CYP2D6. Tricosane also blocks CYP2C19, which means it could cause more interactions with other drugs, but all the three acts as CYP1A2 inhibitors. Although none of the molecules are renal OCT2 substrates, tricosane has the highest predicted total clearance (log 1.924 ml/min/kg) in terms of excretion, indicating quick systemic elimination. No chemicals are likely to be hepatotoxic or AMES toxic from a toxicological perspective. All chemicals exhibit skin sensitivity, indicating a potential for cutaneous reactions. With LD_50_ acute oral toxicity values ranging between 1.683 and 2.062 mol/kg, the compounds are comparable. Carvacrol has the highest chronic toxicity, with the maximum calculated by LOAEL (log 2.308 mg/kg bw/day), reflecting an enhanced long-term safety profile. Expected hERG II suppression for tricosane is also potentially a cardiac risk.

**Table 6:** ADMET parameters of carvacrol, menthol, and tricosane

Property	Model Name	Carvacrol	Menthol	Tricosane
Absorption	Water solubility (log mol/L)	− 3	− 2.487	− 7.539
	Caco2 permeability (log Papp in 10^–6^ cm/s)	1.379	1.362	1.288
	Intestinal absorption (human) (% Absorbed)	95.56	96.545	90.626
	Skin Permeability (log Kp)	− 1.662	− 1.974	− 2.735
	P-glycoprotein substrate	Yes	Yes	Yes
	P-glycoprotein I/II inhibitor	No	No	No/Yes
Distribution	VDss (human) (log L/ kg)	0.034	0.321	1.206
	Fraction unbound (human) (Fu)	0.311	0.44	0
	BBB permeability (log BB)	0.355	0.367	1.173
	CNS permeability (log PS)	− 1.168	− 1.926	− 0.37
Metabolism	CYP2D6/ CYP3A4 substrate	No	No	Yes/No
	CYP1A2 inhibitor	Yes	Yes	Yes
	CYP2C19/ CYP2C9 inhibitor	No	No	Yes/No
	CYP2D6/ CYP3A4 inhibitor	No	No	No
Excretion	Total Clearance (log ml/min/kg)	0.23	0.97	1.924
	Renal OCT2 substrate	No	No	No
Toxicity	AMES toxicity	No	No	No
	Max. tolerated dose (human) (log mg/kg/day)	1.784	1.32	1.6
	hERG I/II inhibitor	No	No	No/Yes
	Oral Rat Acute Toxicity (LD50) (mol/ kg)	2.014	2.062	1.683
	Oral Rat Chronic Toxicity (LOAEL) (log mg/kg_bw/day)	2.308	1.852	1.146
	Hepatotoxicity	No	No	No
	Skin Sensitisation	Yes	Yes	Yes
	T.Pyriformis toxicity (log ug/L)	0.994	0.127	0.851
	Minnow toxicity (log mM)	0.92	1.246	− 4.551

In summary, tricosane exhibits superior tissue distribution and clearance, however, it may have drawbacks due to its low solubility, enzyme interactions, and cardiotoxicity, even though each of the three compounds has good absorption and non-mutagenicity. More balanced ADMET profiles of menthol and carvacrol make them comparatively safer candidates for drug development. However, as the chemicals investigated exhibit good anti-tyrosinase activity, skin sensitivity should be thoroughly investigated for skin applications to avoid any potential skin reactions.

## Discussion

The present study assessed the biological properties of Moroccan CEEO, widely used in traditional medicine, as well as their main volatile compounds, namely carvacrol, menthol, and tricosane, by combining both in vitro and i*n silico* approaches. Overall, the chemical composition of our CEEO is consistent qualitatively as well as quantitatively with those obtained from Croatian CEEO containing high levels of menthol (7%), carvacrol (6.1%), and tricosane (6.8%) (Jerković et al. 2012). Nevertheless, the most important components of Serbian CEEO were neophytadiene isomer III (10.1%), carvacrol (7.9%), p-camphene (5.6%), hexadecanoic acid (4.9%), and thymol (4.2%) (Jovanović et al. 2009). In a recent investigation, the Algerian CEEO revealed β-copaen-4α-ol (38.41%), manool (8.2%), and carvacrol (6.43%) as the main chemotypes (Soltani et al. 2023). These disparate findings could be ascribed to multiple factors, i.e., origin, cultivar, stage of plant development, harvest time, latitude, extraction method, and others (Bouyahya et al. 2019a, b; Bozova et al. 2024; El-Jalel et al. 2018; Gölükcü et al. 2024; Ramsey et al. 2020; Russo et al. 2013; Song et al. 2023; Tammar et al. 2019).

As previously reviewed by El Menyiy et al., CEEO was found to exhibit marked antibacterial activity toward both Gram-positive and negative bacteria (El Menyiy et al. 2021). Hence, Kumarasamy et al. revealed that *S. aureus* was the most sensitive strain (Kumarasamy et al. 2002) and Šiler et al. corroborated these findings by revealing also that *S. aureus* and *E. coli* were the most susceptible strains to the extracts of *C. erythraea* (Šiler et al. 2014). Božunović et al. showed that the growth of *L. monocytogenes* was also inhibited. Besides, *C. erythraea* phenolic extracts exerted promising effect, especially against *S. aureus* (Božunović et al. 2018). The less sensitivity of Gram-negative strains could be ascribed to phospholipids and lipopolysaccharides present in their membrane that ensure protection against the external molecules (Benali, Bouyahya, et al., 2020a, b; Cosentino et al. 1999; Sokovic et al. 2008). It is also noticeable that the three molecules used separately showed more efficiency than the CEEO toward the four strains. *E. coli* was resistant to CEEO, while it was susceptible to the three molecules (IZD > 14 mm). Indeed, the efficiency of carvacrol toward *E. coli* appears to be attributed to its ability to stimulate cytoplasmic membrane permeability and depolarization (Xu et al. 2008) by augmenting permeability, lowering membrane integrity, blocking the quorum sensing process, and causing interference with signaling pathways (Ultee et al. 1999). As recently reviewed by Zhao et al., multiple studies revealed that peppermint EO possesses promising antimicrobial activity toward a vast range of bacterial strains, mainly due to the high amounts of menthol (Zhao et al. 2022). Trombetta et al. mentioned that menthol leads to the disruption of the plasma membrane’s lipid fraction, which modifies the membrane permeability and allows the efflux of intracellular materials (Trombetta et al. 2005). Otherwise, Qiu et al. found that menthol hampered the expression of virulence genes in *S. aureus* (Qiu et al. 2011). Menthol seems to be the main compound responsible for the bacterial growth inhibitory activity of *Mentha piperita* EOs (Işcan et al. 2002). In addition, menthol combined with known antibiotics resulted in synergy (Inouye et al. 2001; Kamatou et al. 2013).

The antioxidant activity of CEEO has been documented in a several previous studies via methods such as ABTS, FRAP, DPPH, Thiobarbituric Acid Reactive Substances (TBARS), Total Antioxidant Capacity (TAC), and Nitric Oxide (NO) (El Menyiy et al. 2021). Our earlier study revealed that CEEO had stronger antioxidant activity at three phenological stages using FRAP, DPPH, and ABTS assays, with EOs from the flowering stage as the most efficient one (Bouyahya et al. 2019a, b). As regards the pure compounds, it appears that their structures impact antioxidant efficacy. Diterpene derivatives were found to be the most effective antioxidants; the location of the double bond in the six-carbon ring is crucial. The number of hydroxyl groups and the configuration of substitutes on the aromatic ring also influenced the antioxidant power (Martignago et al. 2023; Touhtouh et al. 2023). Samarghandian et al. proved in vivo that carvacrol supplementation helped reduce damage caused by oxidative stress to the brain, liver, and kidney (Samarghandian et al. 2016). Additionally, encapsulation of carvacrol lowered malondialdehyde levels and oxidative stress in a rodent model (Carvalho et al. 2020). In addition, menthol-rich EOs were found to be strong antioxidants (Hoseini et al. 2020; Jirovetz et al. 2009). Rozza et al. found that menthol (0.5%) stimulated the action of superoxide dismutase (SOD), glutathione reductase (GR), and glutathione peroxidase (GPx), as well as raised the level of glutathione (GSH) (Rozza et al. 2021).

The comparisons of the anti-enzymatic effect of CEEO and its main compounds with standard molecules such as acarbose offer details about their relative effectiveness and also suggest their potential applications in diabetes management. This could have profound implications for the development of therapies targeting the control of blood sugar levels, particularly for individuals suffering from carbohydrate metabolism-related disorders such as diabetes. In vitro investigations showed that CEEO, can effectively inhibit enzymes with IC_50_ values of 31.91 μg/mL for the α-amylase and 56.77 μg/mL for the α-glucosidase (Bouyahya et al. 2019a, b). Other studies, such as those conducted by Đorđević et al. (Đorđević et al. 2019) and Petrović et al. (Petrović et al. 2024), looked at the *C. erythraea* extract and a herbal mixture containing this plant and found that they have notable antidiabetic properties. Reserachers observed a significant improvement in diabetic rats, including increased insulin levels and preservation of pancreatic islet structure and function. Similarly, a mix of different herbs, including *C. erythraea*, was efficient in normalizing blood glucose levels in diabetic animals, surpassing the effects of insulin and glimepiride treatments (Petrović et al. 2024).

Carvacrol has been the subject of numerous research studies targeting its potential in managing diabetes. These investigations have primarily concerned its impact on blood glucose regulation, insulin levels, and its potential influence on the structure and function of critical enzymes and organs associated with diabetes. However, it is crucial to note that while these studies underscore the properties of carvacrol, further research is necessary to validate its efficiency and safety, particularly in the context of diabetes (Aljelehawy et al. 2023). The study of Govindaraju and Arulselvi revealed that carvacrol, found in the EOs of *Coleus aromaticus* leaves, inhibits both α-amylase and α-glucosidase (Govindaraju & Arulselvi 2018). Ezhumalai et al. emphasized the efficacy of combining carvacrol with rosiglitazone in a diabetic mouse model (Ezhumalai et al. 2014). For menthol, the comprehensive study conducted by Muruganathan et al. elucidated the substantial impact of menthol on the metabolic mechanisms involved in diabetes in rats exposed to a streptozotocin-nicotinamide (STZ-NA)-induced diabetes model (Muruganathan et al. 2013). Notably, menthol significantly reduced blood glucose levels and glycosylated hemoglobin (HbA1c), while greatly increasing total hemoglobin, plasma insulin, and hepatic glycogen levels in these rats. Additionally, the levels of liver enzymes participating in glucose metabolism returned to normal, as did the blood markers indicating liver damage (Muruganathan et al. 2013). Otherwise, it has become evident that there is a scarcity of research specifically focusing on the antidiabetic activity of tricosane. This gap in the scientific literature underscores the lack of in-depth studies or research on the antidiabetic properties of this substance. Consequently, our research proves to be the first to begin a detailed and specific exploration of the potential effects of tricosane as an antidiabetic agent. This originality illustrates the importance of our study in filling this knowledge gap and providing crucial data in the field of diabetes management research.

Many studies on the anti-tyrosinase effect of *C. erythraea* have generated considerable interest in its potential for treating skin pigmentation issues. Our earlier research showed that CEEO significantly hampered the activity of tyrosinase, particularly during the flowering and post-flowering stages (Bouyahya et al. 2019a, b). The study by Zam et al. explored the anti-tyrosinase activity of *C. erythraea* by improving the way phenolic and antioxidant compounds are extracted (Zam et al. 2021). Investigations evaluating the impact of carvacrol on the activity of the tyrosinase enzyme revealed significant effectiveness. The study of Ashraf and his team by synthesizing carvacrol derivatives (compounds 4a–f and 6a–d) comprising benzoic acid and cinnamic acid residues showed that the derivative 6c is particularly effective (IC_50_ of 0.0167 μM), greatly surpassing the reference kojic acid (IC_50_ = 16.69 μM) (Ashraf et al. 2017). Jeon et al. explored the regulatory power of carvacrol from *Thymus vulgaris* oils on tyrosinase (Jeon et al. 2018). This molecule regulated the expression of key proteins involved in melanogenesis, including tyrosinase, CREB, and TRP-1, as well as influencing MITF protein degradation via activation of ERK kinase (Jeon et al. 2018). Brotzman et al. modified carvacrol into alkyl 4-oxobutanoate derivatives to evaluate their inhibitory effect on tyrosinase (Brotzman et al. 2019). These derivatives exhibited inhibitory activity, demonstrating a structural correlation. Another study tested carvacrol from *Origanum* EOs, revealing an inhibition of 56.55% ± 3.10% (El Khoury et al. 2019). This underscores the significant capacity of carvacrol to inhibit the tyrosinase enzyme, offering promising prospects. Overall, these studies suggest that carvacrol and its derivatives might be useful in creating products that could control skin pigmentation, by affecting especially tyrosinase activity. Ultimately, it is important to emphasize that, despite the extensive scientific literature on organic compounds and their effects on tyrosinase, studies focusing on tricosane and menthol remain remarkably limited. Overall, tricosane has superior tissue distribution and clearance; nonetheless, it may present disadvantages due to its limited solubility, enzyme interactions, and cardiotoxicity, despite all three compounds demonstrating favorable absorption and non-mutagenicity. The more balanced ADMET profiles of menthol and carvacrol render them safer options for medication development.

The current study indicates that the biological activities of CEEO are much greater than those of its major components (menthol, carvacrol, and tricosane). This could be attributed to two primary factors: (i) The biological effects of EOs are mostly ascribed to the distinct activity of their major components and their interactions, particularly synergistic and/or additive effects (Basavegowda & Baek 2021). The synergistic effects justify the efficiency of complete EOs instead of separate components (Angane et al. 2024). However, a rational combinations of the most active compounds could help enhancing health benefits, and understanding how they work together to guide drug development and combination therapy (Lebrazi et al. 2025). (ii) Although major compounds get the spotlight, the minor ones, can significantly influence the overall effects of EOs, often augmenting or altering the impacts of the major components (Meenu et al. 2023). Minor compounds, despite their smaller concentrations relative to majors, can have substantial biological impacts (Bassolé & Juliani 2012; de Sousa et al. 2023). Minor chemicals could clarify the overall efficacy of EOs in comparison to their major compounds (Meenu et al. 2023). Additionally, they often play helpful roles that boost or improve the effectiveness of the major components (Angane et al. 2022; Miladinović et al. 2021). Their distinctive pharmacological actions may impart unusual therapeutic qualities, as some “minor” substances exhibit significant biological effects even at low concentrations. The role of minor compounds is becoming more recognized as important for the overall effectiveness of EOs, as they may affect the absorption, distribution, metabolism, and excretion (ADME) profiles (Ben Miri 2025). Researchers are conducting more studies on their synergistic activities, particularly using omics methods and cell signaling tests.

To sum up, these pioneering results provide an essential starting point for future research to gain a better comprehension of the potentialities of these compounds, paving the way for new applications and for the development of innovative cosmetic and pharmaceutical products.

## Conclusion and perspectives

The study emphasized that the main phytochemicals present in the CEEO exhibit remarkable antioxidant, antibacterial, anti-diabetic, and dermatoprotective properties. Overall, the measured diameters of inhibition zones revealed that CEEO, carvacrol, menthol, and tricosane showed potent efficacy toward both Gram-positive strains, while only carvacrol showed potency toward both Gram-negative strains. In the DPPH, ABTS, and FRAP assays, CEEO, carvacrol, and menthol demonstrated significantly greater antioxidant power than Trolox. Regarding the antidiabetic ability, carvacrol, menthol, and tricosane were much better at inhibiting α-glucosidase than acarbose, but only CEEO showed a strong effect on α-glucosidase and α-amylase that was better than acarbose. The ability to inhibit tyrosinase showed that CEEO and tricosane were more effective than quercetin while carvacrol and menthol had similar results. Neither CEEO nor carvacrol, menthol, and tricosane inhibited elastase. These findings suggest considerable therapeutic potential for these bioactive molecules, whereas it is imperative to deeply investigate the biopharmaceutical and pharmacokinetic features especially for skin application as in silico ADMET assessment revealed potential skin reactions. Furthermore, in vivo studies are essential as they demonstrate biological effects at the physiological level, providing crucial data for potential clinical applications.

## Data Availability

Data is provided within the manuscript.
